# CHANGES IN PERSONALITY TRAITS AND SOCIAL SUPPORT BEFORE AND AFTER RETIREMENT

**DOI:** 10.1093/geroni/igac059.1469

**Published:** 2022-12-20

**Authors:** Gabrielle Pfund, Patrick Hill, Mathias Allemand, Marie Kivi, Anne Ingeborg Berg, Valgeier Thorvaldsson, Isabelle Hansson

**Affiliations:** Northwestern University, Chicago, Illinois, United States; Washington University in St. Louis, St. Louis, Missouri, United States; University of Zurich, Zurich, Zurich, Switzerland; University of Gothenburg, Gothenburg, Vastra Gotaland, Sweden; University of Gothenburg, Gothenburg, Vastra Gotaland, Sweden; University of Gothenburg, Gothenburg, Vastra Gotaland, Sweden; University of Gothenburg, Gothenburg, Vastra Gotaland, Sweden

## Abstract

Aging is tied to transitions in occupational, social, and personal contexts, which can have implications for changes in who one is and how connected they feel to others. The current study uses data from 5,844 older adults (Ages: 60-66) with six annual reports on the Big Five personality traits and three distinct social support types (family, friends, relationships) to investigate how personality and social support change together, and the role retirement plays in these changes. Random intercept-cross lagged panel models were conducted to evaluate the associated changes in each trait and social support type while accounting for the time-varying covariate of retirement status. Higher social support across all relationship types was associated with higher agreeableness, extraversion, and conscientiousness and lower neuroticism both between- and within-person. Furthermore, entering retirement predicted an increase in social support with friends and relationship partners, but no consistent change in any of the personality traits.

